# Supplementary material for: Empirical methods for controlling false positives and estimating confidence in ChIP-Seq peaks
Source: BMC Bioinformatics. 2008 Dec 5;9:523. doi: 10.1186/1471-2105-9-523 (PMC2628906; doi:10.1186/1471-2105-9-523)
Supplement: Additional File 1 — A variety of html documents from the USeq web site detailing the available applications, their best usage, output file type descriptions, command line menus, etc. [file 1471-2105-9-523-S1.zip › USeqUserGuides/JavaDoc.zip!/JavaDoc/bioroot/SearchBean.html]

SearchBean


|  |  |  |
| --- | --- | --- |
| **Overview** \| **Package** \| **Class** \| **Use** \| **Tree** \| **Deprecated** \| **Index** \| **Help** | |  |
| **PREV CLASS**   **NEXT CLASS** | **FRAMES**    **NO FRAMES**     **All Classes** |  |
| SUMMARY: NESTED \| FIELD \| CONSTR \| METHOD | DETAIL: FIELD \| CONSTR \| METHOD |  |


---


## bioroot Class SearchBean

```
java.lang.Object
  bioroot.SearchBean
```

---

``` public class SearchBean extends java.lang.Object ```

Bean to hold info about a search request

**Author:**
:   nix

---

**Constructor Summary**

`SearchBean()`


| **Method Summary** | |
| --- | --- |
| `java.lang.String[]` | `fetchSearchColumns()`             Extracts which columns to search for given group selections and field selections |
| `java.lang.String` | `getBlastResults()` |
| `java.lang.String` | `getFieldGroup()` |
| `java.lang.String[]` | `getFields()` |
| `java.lang.String` | `getMatching()` |
| `java.lang.String` | `getMessages()` |
| `java.lang.String` | `getSearchFor()` |
| `java.lang.String` | `getTableName()` |
| `java.lang.String[]` | `processSearchForField()` |
| `SearchHit[]` | `searchForSimilarMatches(java.lang.String[] columns, java.lang.String[] words, DBUtil util, boolean searchForWordBoundaryMatches, UserBean userBean)`             Searches a list of columns for a set of words, case insensitive, %word% matches. |
| `void` | `setBlastResults(java.lang.String blastResults)` |
| `void` | `setFieldGroup(java.lang.String string)` |
| `void` | `setFields(java.lang.String[] strings)` |
| `void` | `setMatching(java.lang.String string)` |
| `void` | `setMessages(java.lang.String string)` |
| `void` | `setSearchFor(java.lang.String string)` |
| `void` | `setTableName(java.lang.String string)` |

**Methods inherited from class java.lang.Object**

`equals, getClass, hashCode, notify, notifyAll, toString, wait, wait, wait`

**Constructor Detail**

### SearchBean

```
public SearchBean()
```


**Method Detail**

### searchForSimilarMatches

```
public SearchHit[] searchForSimilarMatches(java.lang.String[] columns,
                                           java.lang.String[] words,
                                           DBUtil util,
                                           boolean searchForWordBoundaryMatches,
                                           UserBean userBean)
```

:   Searches a list of columns for a set of words, case insensitive, %word% matches. Uses UserBean to limit search to visible items,
    ie those owned by them

---


### fetchSearchColumns

```
public java.lang.String[] fetchSearchColumns()
```

:   Extracts which columns to search for given group selections and field selections

---


### processSearchForField

```
public java.lang.String[] processSearchForField()
```

---


### getFieldGroup

```
public java.lang.String getFieldGroup()
```

---


### getSearchFor

```
public java.lang.String getSearchFor()
```

---


### setFieldGroup

```
public void setFieldGroup(java.lang.String string)
```

---


### setSearchFor

```
public void setSearchFor(java.lang.String string)
```

---


### getFields

```
public java.lang.String[] getFields()
```

---


### setFields

```
public void setFields(java.lang.String[] strings)
```

---


### getMatching

```
public java.lang.String getMatching()
```

---


### getTableName

```
public java.lang.String getTableName()
```

---


### setMatching

```
public void setMatching(java.lang.String string)
```

---


### setTableName

```
public void setTableName(java.lang.String string)
```

---


### getMessages

```
public java.lang.String getMessages()
```

---


### setMessages

```
public void setMessages(java.lang.String string)
```

---


### getBlastResults

```
public java.lang.String getBlastResults()
```

---


### setBlastResults

```
public void setBlastResults(java.lang.String blastResults)
```


---


|  |  |  |
| --- | --- | --- |
| **Overview** \| **Package** \| **Class** \| **Use** \| **Tree** \| **Deprecated** \| **Index** \| **Help** | |  |
| **PREV CLASS**   **NEXT CLASS** | **FRAMES**    **NO FRAMES**     **All Classes** |  |
| SUMMARY: NESTED \| FIELD \| CONSTR \| METHOD | DETAIL: FIELD \| CONSTR \| METHOD |  |


---
